# Effect of sensor location on continuous intraperitoneal glucose sensing in an animal model

**DOI:** 10.1371/journal.pone.0205447

**Published:** 2018-10-09

**Authors:** Marte Kierulf Åm, Konstanze Kölle, Anders Lyngvi Fougner, Ilze Dirnena-Fusini, Patrick Christian Bösch, Reinold Ellingsen, Dag Roar Hjelme, Øyvind Stavdahl, Sven Magnus Carlsen, Sverre Christian Christiansen

**Affiliations:** 1 Department of Clinical and Molecular Medicine, Faculty of Medicine and Health Sciences, Norwegian University of Science and Technology (NTNU), Trondheim, Norway; 2 Department of Endocrinology, St Olav’s Hospital, Trondheim, Norway; 3 Department of Engineering Cybernetics, Faculty of Information Technology and Electrical Engineering, Norwegian University of Science and Technology (NTNU), Trondheim, Norway; 4 Department of Electronic Systems, Faculty of Information Technology and Electrical Engineering, Norwegian University of Science and Technology (NTNU), Trondheim, Norway; Medical University of Vienna, AUSTRIA

## Abstract

**Background:**

In diabetes research, the development of the artificial pancreas has been a major topic since continuous glucose monitoring became available in the early 2000’s. A prerequisite for an artificial pancreas is fast and reliable glucose sensing. However, subcutaneous continuous glucose monitoring carries the disadvantage of slow dynamics. As an alternative, we explored continuous glucose sensing in the peritoneal space, and investigated potential spatial differences in glucose dynamics within the peritoneal cavity. As a secondary outcome, we compared the glucose dynamics in the peritoneal space to the subcutaneous tissue.

**Material and methods:**

Eight-hour experiments were conducted on 12 anesthetised non-diabetic pigs. Four commercially available amperometric glucose sensors (FreeStyle Libre, Abbott Diabetes Care Ltd., Witney, UK) were inserted in four different locations of the peritoneal cavity and two sensors were inserted in the subcutaneous tissue. Meals were simulated by intravenous infusions of glucose, and frequent arterial blood and intraperitoneal fluid samples were collected for glucose reference.

**Results:**

No significant differences were discovered in glucose dynamics between the four quadrants of the peritoneal cavity. The intraperitoneal sensors responded faster to the glucose excursions than the subcutaneous sensors, and the time delay was significantly smaller for the intraperitoneal sensors, but we did not find significant results when comparing the other dynamic parameters.

## Introduction

Achieving tight glucose control in diabetes mellitus type 1 (DM1) treatment is a challenge, but crucial in preventing hyperglycaemia-related late complications. However, tighter glucose control is usually accompanied by an increased incidence of severe hypoglycaemia [1, 2]. In addition, patients face the burdens of constant self-surveillance of blood glucose levels, careful planning of exercise and carbohydrate consumption and deciding on dose and delivery of insulin. Fully automatic closed-loop delivery of insulin, i.e. a so-called artificial pancreas (AP), has the potential to revolutionise the way we treat diabetes, removing some of these burdens [3–5]. A well-functioning AP should mimic a healthy pancreas with regard to glucose regulatory function and keep the patients’ blood glucose levels within the narrow safe range. Ideally, an AP obtains the glucose values seen in people without diabetes, thus providing DM1 patients with improved quality of life and longer life expectancy.

The majority of research on AP targets the subcutaneous (SC) tissue as the site for continuous glucose monitoring (CGM) and insulin delivery, the so-called “double SC approach”. Several clinical studies have explored the feasibility of this approach under free-living conditions [6, 7]. In 2016, the first hybrid AP was approved by the U.S. Food & Drug Administration (FDA) [8]. In contrast to a fully automatic closed-loop system, a hybrid AP requires the patients to administer pre-meal insulin boluses themselves [8].

Subcutaneous glucose measurements carry certain limitations, mainly due to slow glucose dynamics [9, 10]. At least 6–7 minutes are needed to transport glucose from the lumen of the capillaries to the SC interstitium [11, 12]. In addition, glucose dynamics in the SC tissue varies significantly between patients [13], and the performance of the CGM sensor is influenced by several factors; such as mechanical pressure [14–16], micro-haemorrhages at sensor site [17], certain drug interactions [18], temperature, fluctuations in tissue perfusion [19, 20] and local foreign body reaction [15, 21]. In addition to physiological factors, the sensor has its own internal dynamics, adding up to the total latency in CGM [10]. Both the delay and the variable dynamics make the SC tissue insufficient for glucose sensing in an AP. This paper uses the definitions of time delay, time constant etc. as earlier described by Stavdahl et al. [10].

Given the delays in SC CGM, the use of the intraperitoneal (IP) space for CGM has been proposed. The glucose dynamics in the IP space has been shown to be fast [22–24]. The IP space also has other advantages compared to the SC tissue, providing a more mechanically and thermally stable environment.

To optimize the potential of a double IP artificial pancreas, it is important that the glucose sensing element be placed at the most appropriate site. To our knowledge potential differences in glucose dynamics within the peritoneal cavity has not previously been studied. The main aim of this study was therefore to explore and compare the glucose dynamics in four different locations within the peritoneal cavity of anesthetised domestic pigs during sessions of IV glucose infusions. Secondly, we compared the performance of IP CGM to SC CGM. In order to resemble normal physiological glucose excursions, glucose challenges were infused as simulated meals of 30 minutes duration, rather than boluses.

## Materials and methods

### Ethical approval

The animal experiments were approved by the Norwegian Food Safety Authority (FOTS numbers 8606 and 12948), and was in accordance with «The Norwegian Regulation on Animal Experimentation» and «Directive 2010/63/EU on the protection of animals used for scientific purposes».

### Animals and animal handling

Between September 2016 and October 2017, twelve juvenile, non-diabetic farm pigs of both genders (1 male, 11 females) weighing 31–44 kg, were brought to the animal research facility approximately one week prior to experiments and acclimatised to the staff and new environment. They were housed together in a common pen, in groups of two or three whenever possible, provided wood chips as nesting material and toys to keep them occupied. The lighting condition was standardised with a 16 hours light period followed by an 8 hours dark period. They were fed standard commercial growth feed twice a day and provided water *ad libitum*. Food was removed 16 hours before the experiments.

### Anaesthesia

The pigs were premedicated with an intramuscular injection of 4 mg diazepam (Actavis Group, Hafnarfjordur, Iceland), 160 mg azaperone (Eli Lilly Regional Operations GmbH, Austria) and 750 mg ketamine (Pfizer AS, Norway), while in the pen. An aurical vein was cannulated and anaesthesia was induced with intravenous (IV) injections of 1 mg atropine (Takeda AS, Asker, Norway), 150–250 μg fentanyl (Actavis Group, Hafnarfjordur, Iceland), 75–125 mg thiopental (VUAB Pharma AS, Roztoky, Czech Republic) and 150–250 mg ketalar (Pfizer AS, Norway).

The pigs were intubated in the lateral position and mechanically ventilated and monitored on an anaesthesia machine (Aisys, GE Healthcare Technologies, Oslo). Anaesthesia was maintained by IV infusion of midazolam (0.5 mg/kg/h) (Accord Healthcare Limited, Middlesex, UK) and fentanyl (7.5 μg/kg/h) (Actavis Group, Hafnarfjordur, Iceland) and by inhalation of isoflurane (0–2%) (Baxter AS, Oslo, Norway). Room temperature was around 20 degrees Celsius. The body temperature of the pigs was monitored, and a heating blanket used when necessary.

The pigs received IV infusion of antibiotics (Cefalotin, Villerton Invest SA, Luxembourg), 2 g immediately after the pigs were anaesthetised and 1 g after 4 hours. Heparin (150 IE) (LEO Pharma A/S, Ballerup, Denmark) was injected in the peritoneal space at the same time points.

Fluid balance was achieved by continuous IV infusion of Ringer’s acetate with individual adjustments to achieve stable blood pressure. To reduce the amount of IP fluid accumulating in the pigs, the amount of Ringer’s acetate was reduced in consecutive experiments, from 5–9 ml/kg/h initially to 2.5 ml/kg/h in the last four pigs. The pigs also received IV fluid through antibiotics, glucose and when the catheters were flushed after every blood sample. Total fluid loss during experiments is not known, but estimates suggest that the pigs were in positive fluid balance, even at the lower infusion regimen.

### Surgical procedure

An intra-arterial line was placed in the left carotid artery for blood sampling and monitoring of physiological parameters and an IV line was placed in the left internal jugular vein for glucose and fluid infusions. Both catheters were inserted through the same cut-down.

The IP sensors were inserted through a 6–8 cm long cranio-caudal incision in the abdominal wall, 2–3 cm caudally to the umbilicus. The bladder was exposed through a small, low laparotomy for the insertion of a bladder catheter. Both cuts were made with a thermocauter to minimise bleeding into the abdominal cavity.

At the end of the experiments, and under full anaesthesia, the pigs were euthanised with an IV overdose of pentobarbital (minimum 100 mg/kg) (pentobarbital NAF, Apotek, Lørenskog, Norway).

One additional pig was used to refine the experimental protocol before the start of the study. The results from this pig are not included in this article.

### Sensors and sensor placement

**Fig 1:**
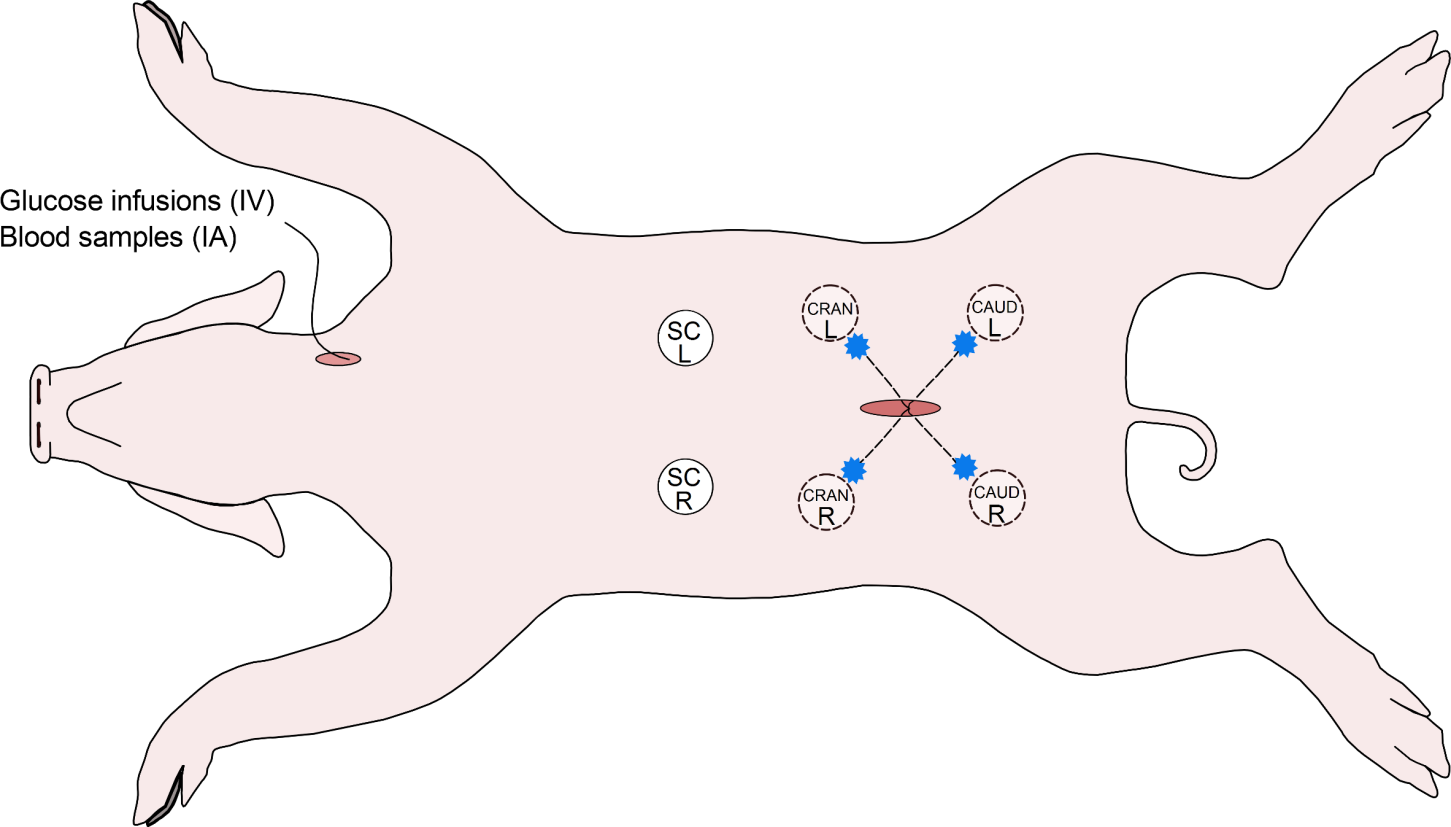
Schematic presentation of sensor placement and sites for intraperitoneal fluid sampling. Blue stars indicate sites for IP fluid sampling. SC = subcutaneous, IP = intraperitoneal, CRAN = cranial, CAUD = caudal, L = left, R = right, IA = intra-arterial, IV = intravenous.

Four unmodified FreeStyle Libre (Abbott Diabetes Care Ltd., Witney, UK) sensors were positioned 10 cm into the abdominal cavity in four different directions, corresponding to the four quadrants of the abdominal cavity as shown in Fig 1. Two FreeStyle Libre sensors were inserted subcutaneously, 5 cm on each side of the ventral medial line at the height of the first pair of nipples, avoiding visual SC blood vessels.

**Fig 2:**
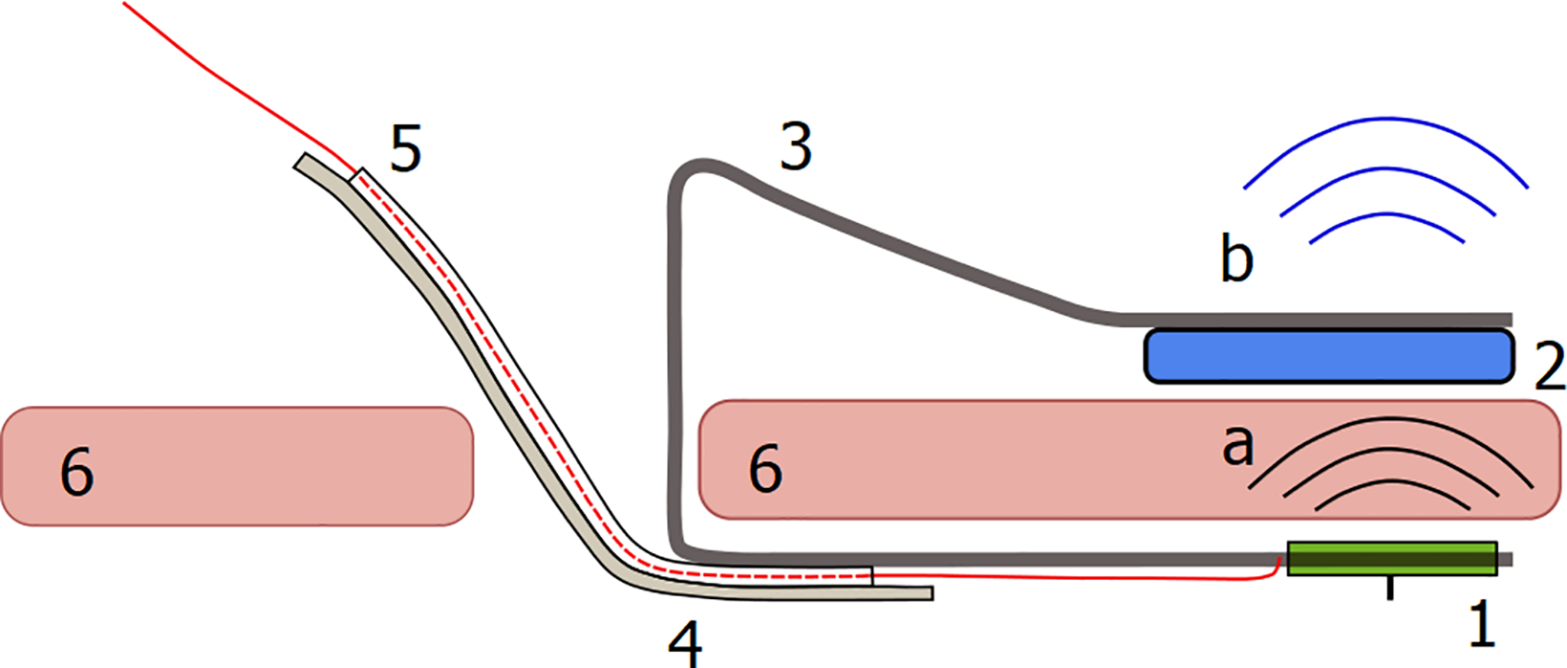
Sketch of retainer for sensor and reading device. The FreeStyle Libre was held in place on the inside of the abdominal wall, while the reading device (LimiTTer) was held tightly to the outside of the abdominal wall [25]. 1 = FreeStyle Libre sensor, 2 = LimiTTer, 3 = Retainer, 4 = IP fluid sampling tube, 5 = Guide wire with guide tube, 6 = Abdominal wall, a = Near-field communication, b = Bluetooth low energy.

Custom made retainers, made of PMMA, were used to hold the sensors in place and also hold the corresponding reading devices (LimiTTer) close enough to the sensors on the outside of the abdominal wall for registration of sensor signals (Fig 2). Data was transferred to an xDrip application for further handling [25]. IP fluid samples from the sensor locations were drawn using the same retainer as described above.

In six of the experiments, two of the four IP-sensors were positioned with the sensor element of the FreeStyle Libre sensor pointing towards the abdominal wall (total 28 sensor recordings). The other sensors were positioned with the sensor element pointing towards the visceral peritoneum (total 84 sensor recordings). Four pigs had one of the SC sensors inserted the day before the start of the experiment (24 hours). The other SC sensors were inserted after the pigs had been anaesthetised and allowed at least 1 hour to settle before the glucose infusions. The IP sensors were submerged in phosphate-buffered saline (Phosphate buffered saline tablet, Sigma-Aldrich Co., St. Louis, USA) with 3mmol/l glucose for approximately 1 hour before insertions. This ensured optimal conditions for the IP sensors in the 1-hour start-up phase, and we could also ensure that the sensor elements were completely exposed to glucose containing fluid, check the set-up functionality, and confirm sensor readings before inserting the sensors into the abdomen of the pigs.

After insertion into the IP space, the sensors were allowed at least 30 minutes to settle before glucose infusions were started. Several in vitro experiments were performed prior to the pig experiments in order to characterise the dynamics of the FreeStyle Libre sensor when subjected to changes in glucose concentration and to test the communication protocols. See publication by Bösch et al. for further details on the in vitro trials of the FreeStyle Sensors [25].

### Glucose infusions

Meals (IV meals) were simulated by stepwise IV infusions of glucose (Glucos B. Braun 200 mg/ml, B. Braun, Melsungen AG, Germany). The total amount of glucose per IV-meal was 245 mg/kg body weight infused over 30 minutes with a glucose rise of 3.5–5.5 mmol/L. IV glucose clamps (lasting 80–110 minutes achieving a glucose rise of 4–6 mmol/L) were performed to calibrate the IP sensors in 8 of the pigs. Table 1 presents the order in which the different glucose infusions were given.

**Table 1:** Order of glucose infusions for all pigs.

Pig ID	First glucose infusion	Second glucose infusion	Third glucose infusion
**2**	Bolus ^a^	IV meal (943 mg/kg)	IV meal (313 mg/kg)
**3**	Bolus ^a^	IV meal (245 mg/kg)	IV meal (245 mg/kg)
**4, 5**	IV meal (245 mg/kg)	IV meal (245 mg/kg)	IV meal (245 mg/kg)
**6, 8, 9**	Clamp	Bolus ^a^	Bolus ^a^
**7, 10, 11, 12, 13**	Clamp	IV meal (245 mg/kg)	IV meal (245 mg/kg)

^a^ Data not included in the study

### Glucose analysis of arterial blood and IP fluid

Arterial blood and IP fluid samples were analysed on a Radiometer ABL 725 blood gas analyser (Radiometer Medical ApS, Brønshøj, Denmark). Blood was collected in heparinised syringes (LEO Pharma A/S, Ballerup, Denmark) and IP fluid was collected in heparinised capillary tubes (Radiometer Medical Aps, Brønshøj, Denmark).

Due to the large quantity of IP fluid samples, some samples needed to be stored on ice for several hours before analysis. Some of them were tested immediately after harvesting and again after being stored for 6 hours. A mean change of + 0.1 mmol/L (SD 0.1 mmol/L) was observed.

### Data processing

The open source devices LimiTTer (LimiTTer by JoernL @ GitHub) were used to relay the data transmitted by the FreeStyle Libre sensors to tablet computers running the open source Android application xDrip (xDrip by stephenblackwasalreadytaken @ GitHub). The LimiTTers were customised to read the FreeStyle Libre sensors approximately every 22 seconds, however, the FreeStyle Libre sensors only update the transmitted glucose values in one-minute intervals. The xDrip app was not customised and was only used to store the transmitted glucose values for later processing and to display the received data in order to supply a mean of monitoring the function of the set-up [22].

**Fig 3:**
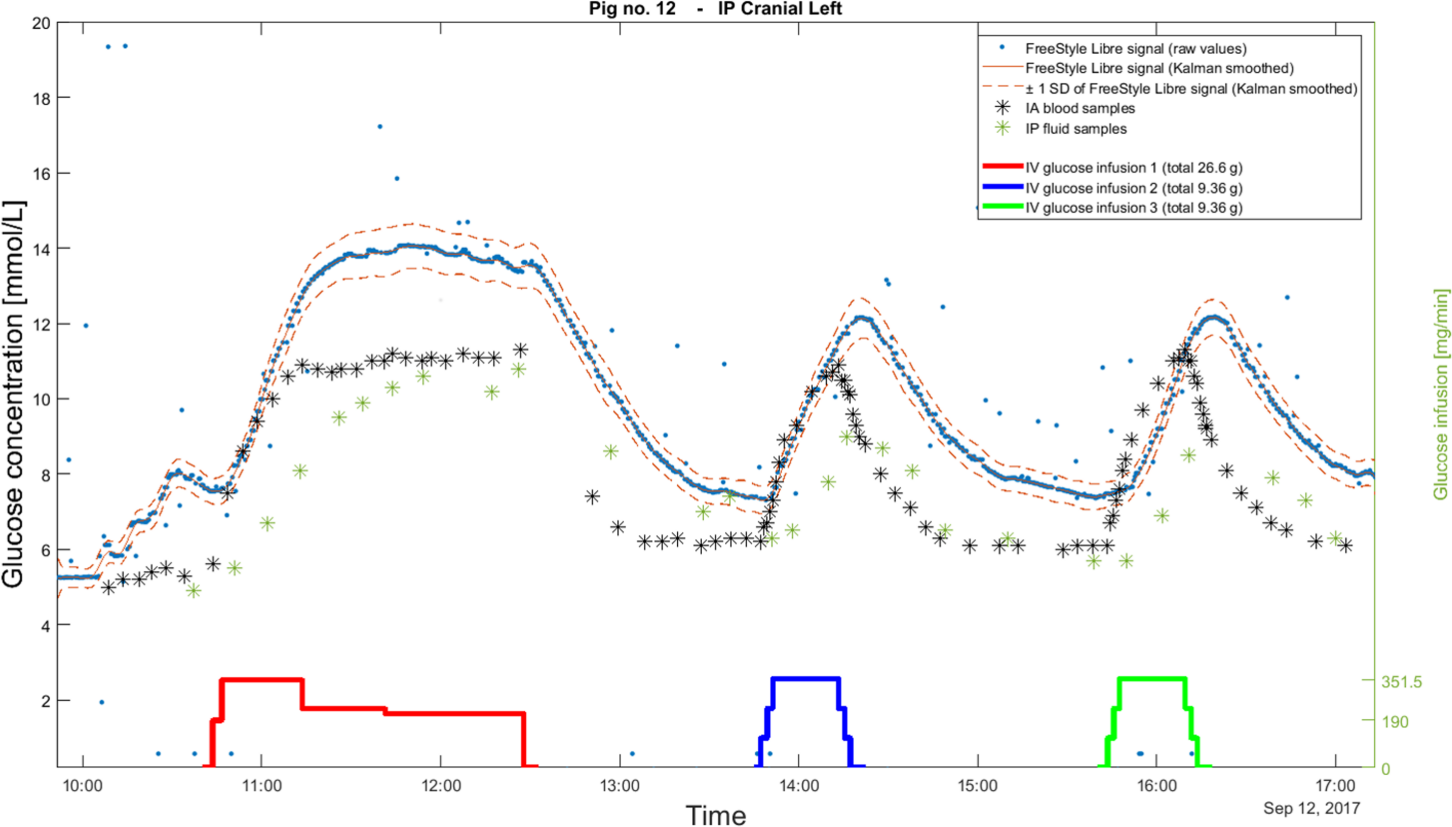
Example of raw data and Kalman smoothed data. The figure shows data from the entire experiment from the cranial left IP sensor in pig 12. The blue points represent raw data read by the LimiTTer, the red solid line represents the Kalman smoothed data and the dashed line represents ± 1 standard deviation of the Kalman smoothed curve. The black stars (*) represent the IA glucose samples and the green stars (*) represent the IP glucose samples of the cranial left IP location. The glucose infusions are seen as solid lines at the bottom part of the graphics.

The raw data from the FreeStyle Libre sensors was denoised using a median filter with a window size of 5 samples, i.e. circa 130 seconds to remove single outliers. The thickness of the abdominal wall caused some transmission noise, and the communication protocol of the LimiTTer was not flawless. The Kalman smoothing method by Staal et al. was then applied to the median-filtered glucose measurements producing interpolated series with a sampling rate of 1 s [26]. The smoother also removes outliers. Fig 3 illustrates how the raw measurements are affected by these methods for denoising and smoothing. Table 2 summarises the setting and tuning parameters.

**Table 2:** Settings and tuning parameters used for Kalman smoothing of FreeStyle Libre data [26].

	FreeStyle Libre
Dynamic model	Model 2: Central-remote rate model
Model parameter	*T*_*d*_ = 600 *s*
Process noise covariance	$Q=\left[\begin{array}{ccc} 0 & 0 & 0\\ 0 & 10\;\Delta t & 0\\ 0 & 0 & 0 \end{array}\right]$ with Δ*t* = 1 *s*
Measurement noise covariance	*R* = (0.83/2)^2^ for *G* ≤ 5.55 mmol/L *R* = (0.15/2)^2^ ⋅ *G*^2^ for *G* > 5.55 mmol/L
Initial covariance	$P_{0}=\left[\begin{array}{ccc} 10 & 0 & 0\\ 0 & 1 & 0\\ 0 & 0 & 1 \end{array}\right]$
Outlier removal	Based on smoothed data (outlierRemoval = 1)

The worst-case variances for self-monitoring blood glucose devices fulfilling ISO 15197:2013 were used.

Intra-arterial blood glucose analysis was performed manually in intervals ranging from 30 seconds to 10 minutes during the glucose infusions, and therefore less frequently than the FreeStyle Libre data. Equally distributed sampling intervals were necessary in order to use the data for system identification. Thus, blood glucose values were processed by a shape-preserving piecewise cubic interpolation to get the same sampling intervals as for the FreeStyle Libre data.

#### Model identification

As described above, continuous glucose sensors were placed in the IP cavity and the SC tissue. The dynamics between the intra-arterial glucose concentrations (*G*_*IA*_) and the sensed glucose concentrations are described by a two-compartment model [22]:

$$\frac{dG_{sens}(t)}{dt}=\frac{1}{\tau }(K∙G_{IA}(t-\Theta )-G_{sens}(t))$$ (1)

with the sensor glucose concentration *G*_*sens*_, the intra-arterial glucose concentration *G*_*IA*_, the time constant *τ*, the time delay *Θ*, and the model gain *K*. The intra-arterial glucose concentration was obtained from arterial blood samples. The sensor glucose concentration is measured by the FreeStyle Libre sensors. Thus, the modelled dynamics include both the physiological dynamics from blood to the sensor site and the internal sensor dynamics.

The time-domain Eq (1) was transferred into the frequency domain where a first-order transfer function with time delay was identified using the System Identification Toolbox in MATLAB (The MathWorks Inc., Natick, Massachusetts, U.S.A.). The optimal input delay was found by repeatedly determining the transfer function with a fixed delay. Time delays between 0 and 900 seconds with 1-second intervals were considered. The time delay that resulted in the lowest mean squared error (MSE) between the modelled and the measured sensor glucose was chosen.

The MATLAB-internal function *tfest* requires that the data starts at 0. Therefore, the stationary glucose value (called baseline in this article) at the beginning of the glucose infusion was subtracted to correct for the offset different from 0. This stationary value was determined for each sensor, site and clamp because it was not guaranteed that the same glucose level was reached between the glucose infusions. The stationary (baseline) values were determined as follows:

1. For intra-arterial glucose values, the sample at the start of the glucose infusion (t = 0 min) and the three preceding samples (over a period of approximately 15 minutes) were averaged, i.e.
  $$G_{IA,stat}=\frac{BGA(t=-15\;min)+BGA(t=-10\;min)+BGA(t=-5min)+BGA(t=0\;min)}{4}.$$
2. For the sensed glucose values (SC and IP), the average over the last 3 minutes preceding the glucose infusion start was taken.

### Statistical analysis

The combined physiological and sensor dynamics were analysed based on the smoothed data from the section Data Processing.

#### Inclusion of sensor recordings

Sensor measurements that fulfilled the following criteria were included in statistical analysis:

1. Stable at beginning of glucose challenge
  The sensor value was stable in the 3 min before the glucose infusion. Stable was defined as a glucose rate of change less than 0.1 mmol/L/min, i.e. if;
  $$\left|\frac{dG_{sens}}{dt}\right|<0.1\frac{mmol}{L∙min}$$
2. Model fit
  The identified model from section Model identification had a goodness of fit of more than 70%.

The following properties were investigated:

1. Time delay of the model identified in section Model identification.
2. Time constant of the model identified in section Model identification.
3. Time to 50% of maximum value from start of glucose challenge.
4. Time to 50% return to baseline levels, counted from start of glucose challenge.

All statistical analyses were conducted in R [27]. Dynamic parameters were analysed using a linear mixed effect analysis with maximum likelihood estimation with the sensor location, the amount of IV fluid infusion and the direction of the sensor element (for IP sensors only) defined as fixed effects. To account for several measurements in each pig, pig ID was defined as a random effect [28]. P-values for pairwise comparisons of the different IP sensor locations and comparison of all IP sensors against all SC sensors, were obtained by t-tests using the Sattertwaite approximation to the effective degrees of freedom, automatically calculated with the lmerTest and lme4 packages in R [28, 29]. The statistical threshold was set to 0.01 to account for multiple comparisons.

## Results

**Fig 4:**
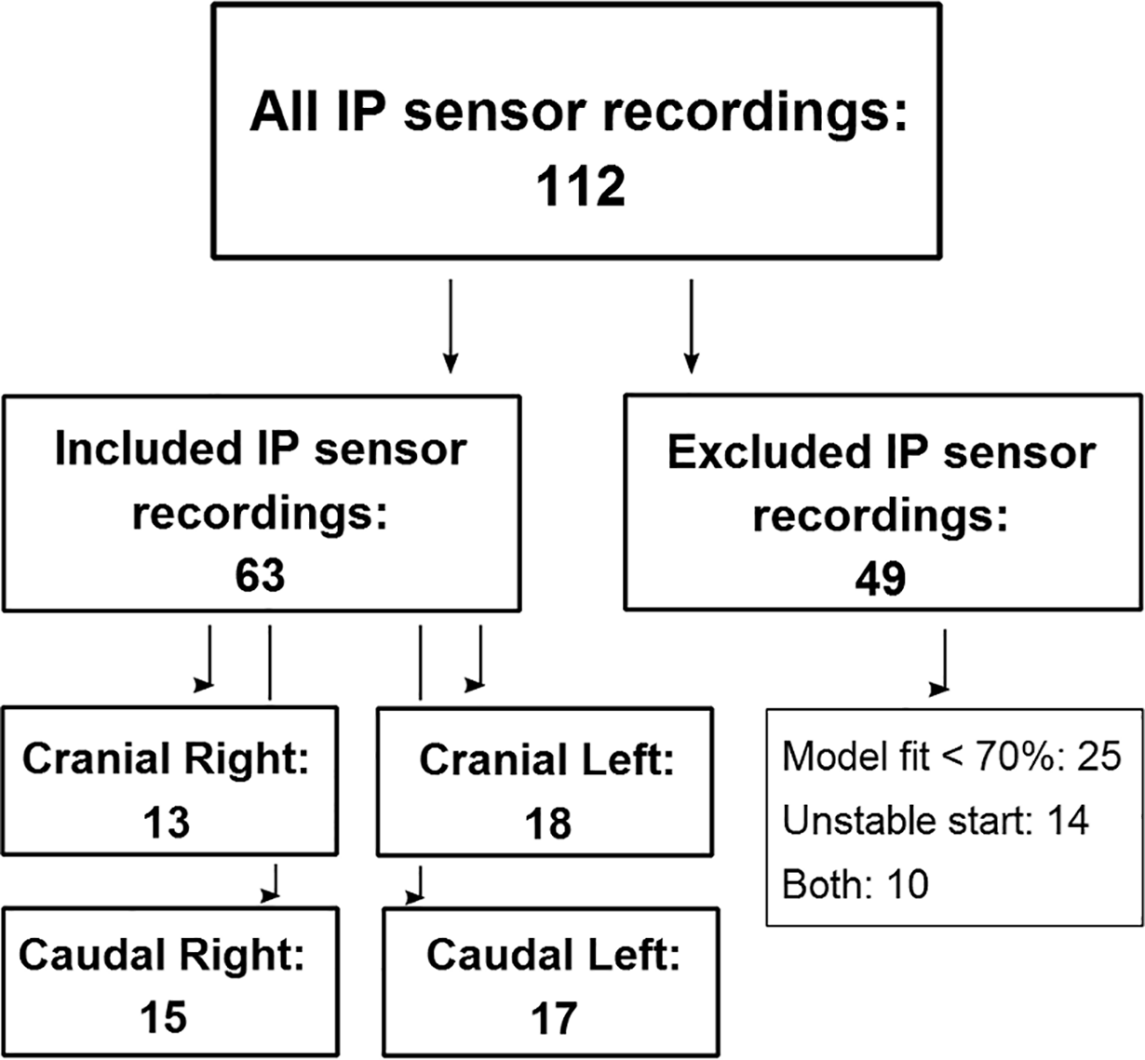
Scheme of included and excluded IP sensor recordings.

**Fig 5:**
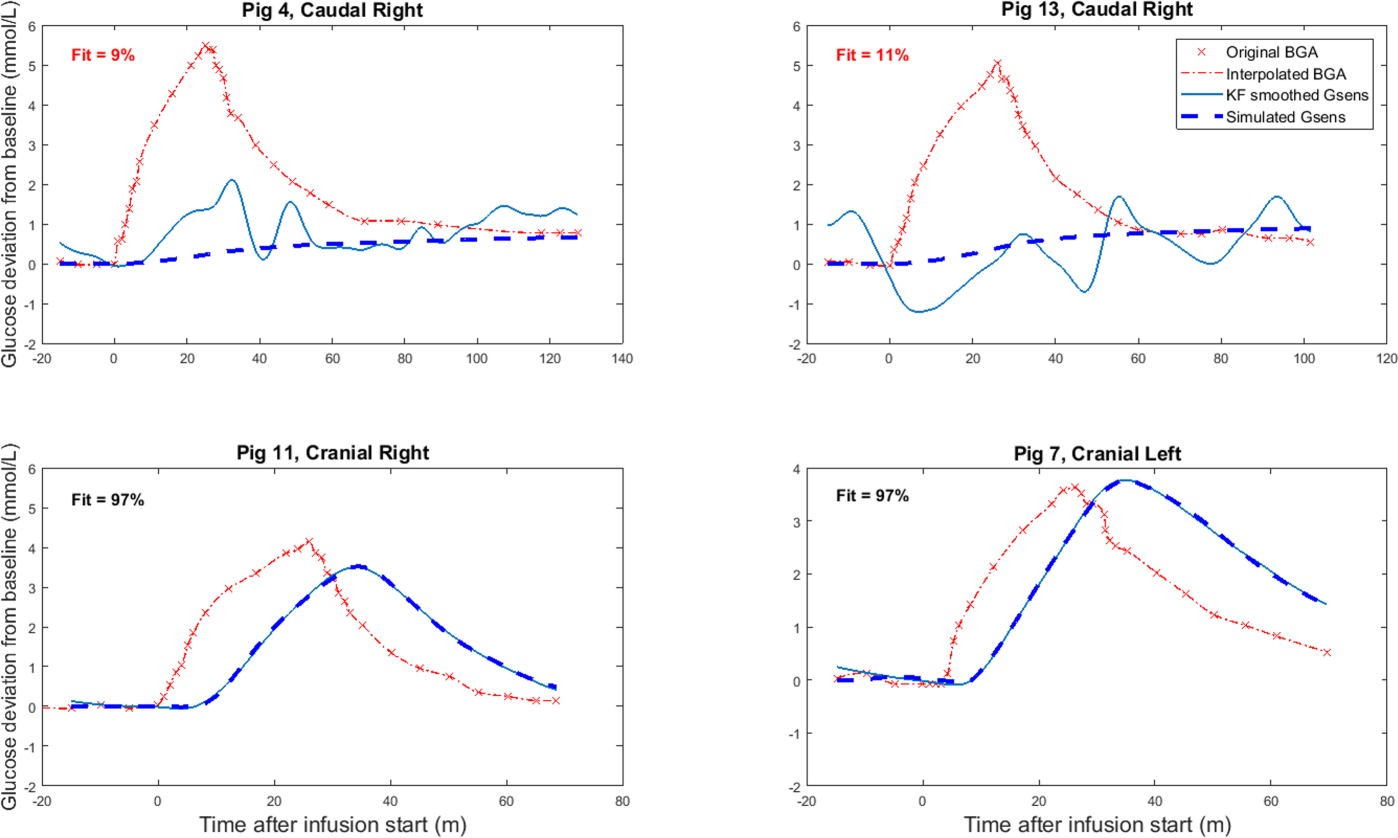
Examples of excluded (top panels) and included (bottom panels) sensor recordings. The red curves represent the intra-arterial blood glucose values, the solid blue lines represent the Kalman smoothed sensor signals and the dashed dark blue lines represent the sensor signals predicted by the model. The percentage of model fit is displayed in the top right corner of each panel. BGA = blood glucose analysis, Gsens = sensor glucose concentration.

168 sensor recordings were recorded from 7 glucose clamps and 20 IV-meals. All SC sensor recordings (56 out of 56) and 56% (63 out of 112) of the IP sensor recordings were included in the statistical analysis (Fig 4). The mean percentage of model fit for the included sensors was 93.4% (SD 4.1%) for the SC sensors and 85.7% (SD 7.5%) for the IP sensors. Fig 5 shows examples of included and excluded sensor recordings based on the criterion of model fit.

**Fig 6:**
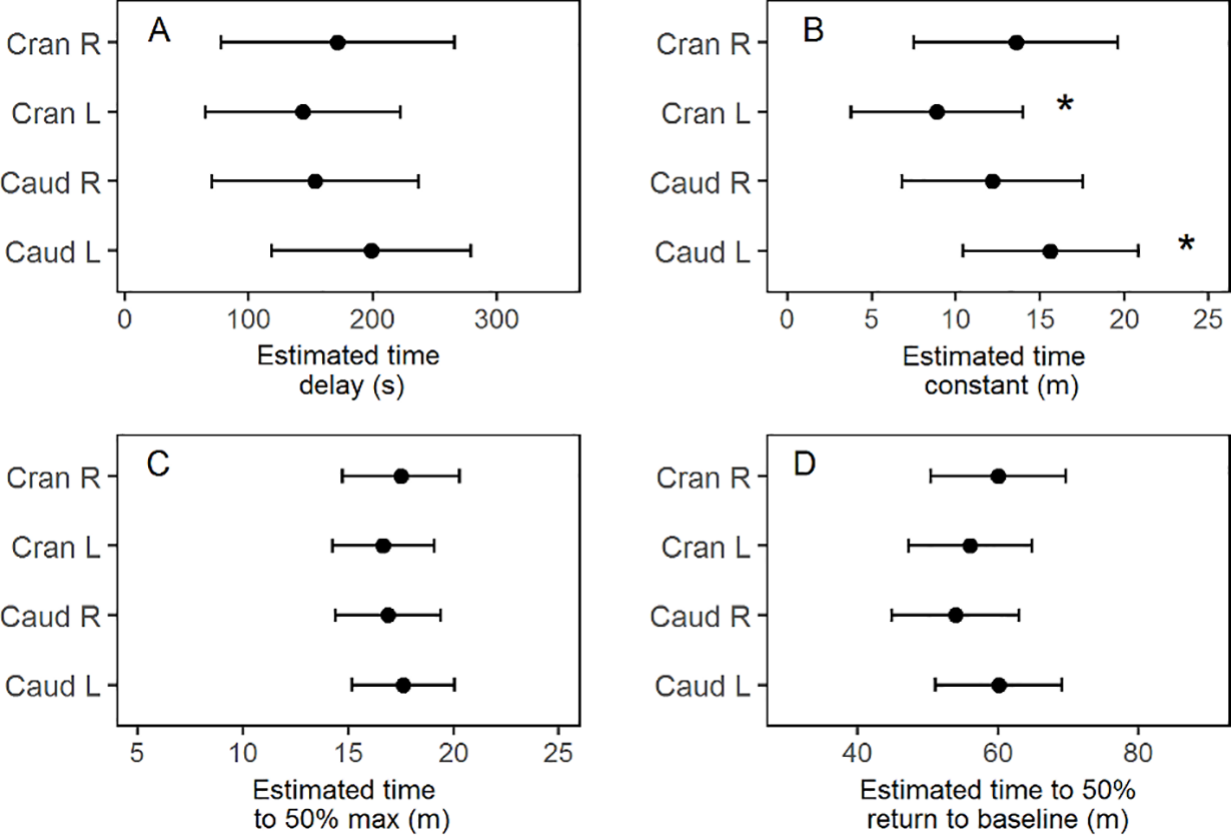
**Point estimates and 95% confidence intervals for time delay (A), time constant (B), time to 50% max (C) and time to 50% decline to baseline level (D) for all IP sensors.** Sensor location is set as fixed effect in the lmerTest-model. * indicates a significant difference (p <0.01).

**Fig 7:**
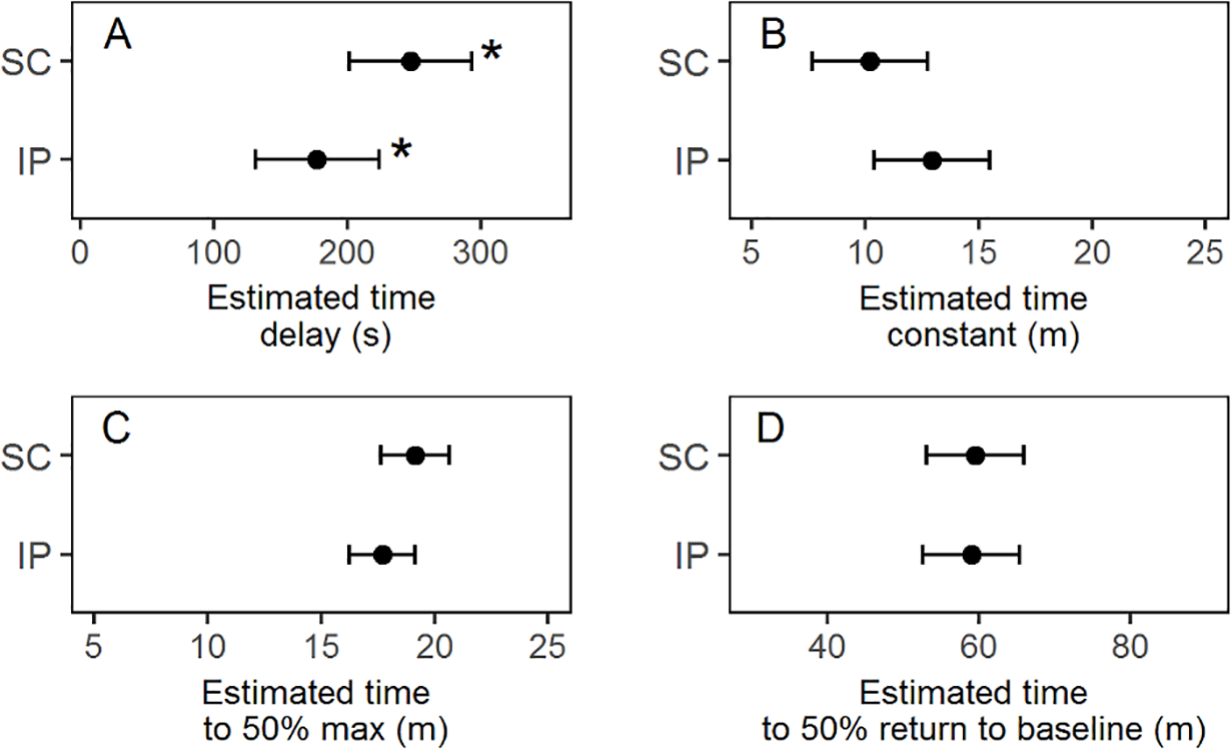
**Point estimates and 95% confidence intervals for time delay (A), time constant (B), time to 50% max (C) and time to 50% decline to baseline level (D) for IP and SC sensors.** The mean parameter values for the IP sensors compared to those for the SC sensors with sensor location (SC or IP) as fixed effect in the lmerTest-model. * indicates a significant difference (p <0.01).

**Fig 8:**
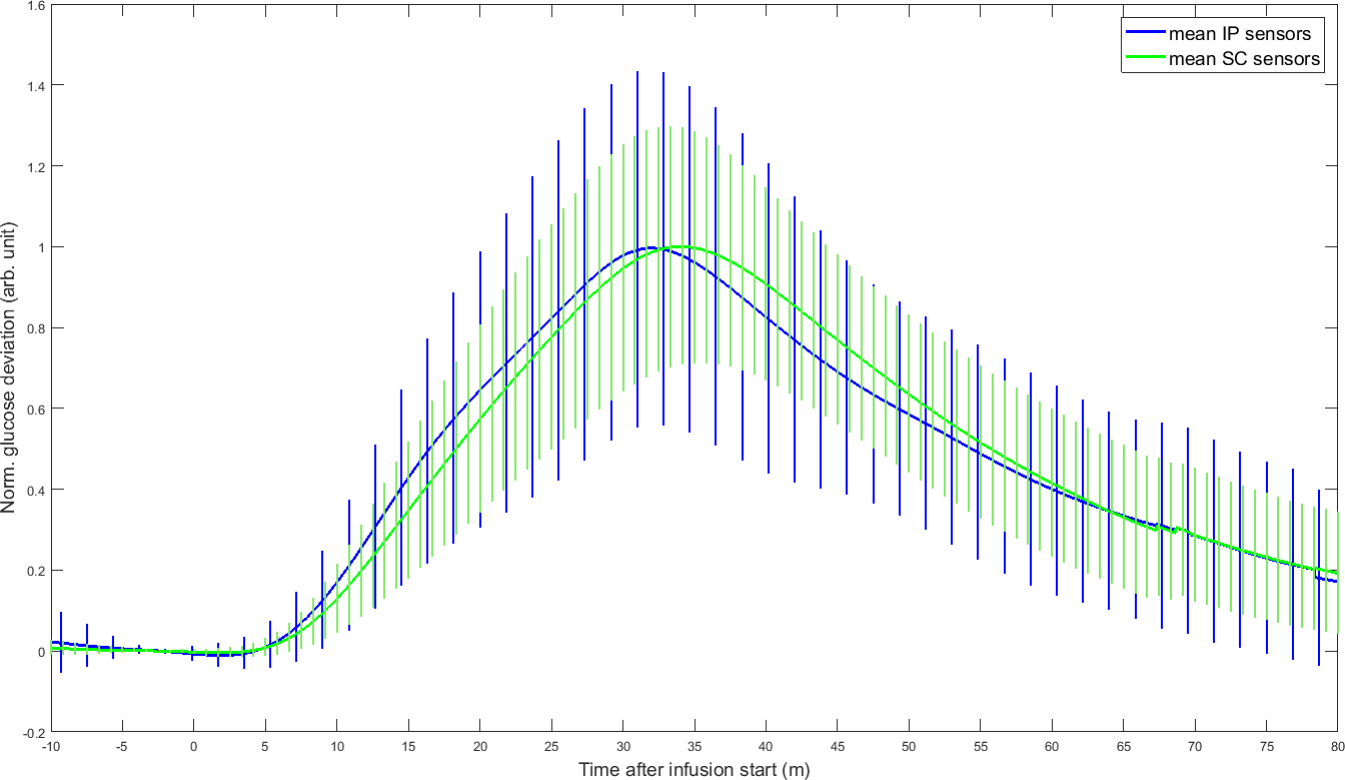
Distribution of sensor recordings from the included IP and SC sensors during IV-meals. The blue curve represents the mean of the normalized IP sensor signals, and the green curve represents the mean of the normalized SC sensor signals. The sensor values were not calibrated and thus do not necessarily show the real glucose values. We therefore compare the normalized glucose deviation from the baseline. The mean was calculated by averaging at each sample over all IP and SC sensors, respectively. The average deviation was then scaled to the range [0, 1]. The same scaling factor was used to normalize all sensor readings from the sensing site under consideration before the standard deviation was determined.

Table 3 presents the mean and standard deviation of the dynamic parameters for the different IP sensor locations, as well as for all SC and IP sensors. The IP cranial left sensors and the IP caudal right sensors seemed to react faster than the IP cranial right and IP caudal left sensors. However, the only pairwise comparison to show a significant difference, was the smaller time constant of IP cranial left sensors compared to the IP caudal left sensors (p = .0075) (Fig 6 and S1 Table). The estimated mean time delay of all IP sensors was significantly smaller than the mean time delay for all SC sensors (p = .0091) (Fig 7 and S2 Table). Comparisons of SC and IP sensors for the other dynamic parameters did not show significant differences (Fig 7 and S2 Table). Fig 8 shows the mean and standard deviation for all included IP and SC sensors during IV-meals, and indicates that the IP sensors react faster than the SC, but also shows a larger variation.

**Table 3:** Summary of results for the four IP sensor locations and mean of the IP and SC sensors.

Sensor location		Mean time delay ± SD (s)^a^	Mean time constant ± SD (min)^a^	Mean model fit ± SD (%)^a^	Mean time to 50% max (min)^b^	Mean time to 50% decline to baseline (min)^b^
**IP Cranial Right**		175.2 ± 139.2	13.2 ± 11.1	85.4 ± 8.0	18.0 ± 4.4	59.1 ± 15.6
**IP Cranial Left**		145.9 ± 127.8	8.6 ± 4.32^1^	85.8 ± 7.3	16.9 ± 2.7	55.8 ± 6.7
**IP Caudal Right**		155.6 ± 139.8	11.9 ± 11.0	82.8 ± 7.6	17.3 ± 3.9	53.0 ± 11.1
**IP Caudal Left**		204.3 ± 160.4	15.6 ± 9.1^1^	87.5 ± 6.4	18.0 ± 2.9	57.9 ± 11.2
**All IP sensors**		170.0 ± 140.8^2^	12.2 ± 9.2	85.5 ± 7.3	17.5 ± 3.4	56.3 ± 11.1
**All SC sensors**		241.1 ± 149.9^2^	9.5 ± 5.3	91.0 ± 6.5	19.0 ± 2.7	57.2 ± 10.1

^a^ Calculations are done on identified model parameters using both glucose clamps and IV meals.

^b^ Calculations done on Kalman smoothed data, exclusively from IV meals.

^1^ Time constant of IP cranial left significantly smaller than IP caudal left, p = .0075.

^2^ Time delay of IP sensors significantly smaller than SC sensors, p = .0091.

**Fig 9:**
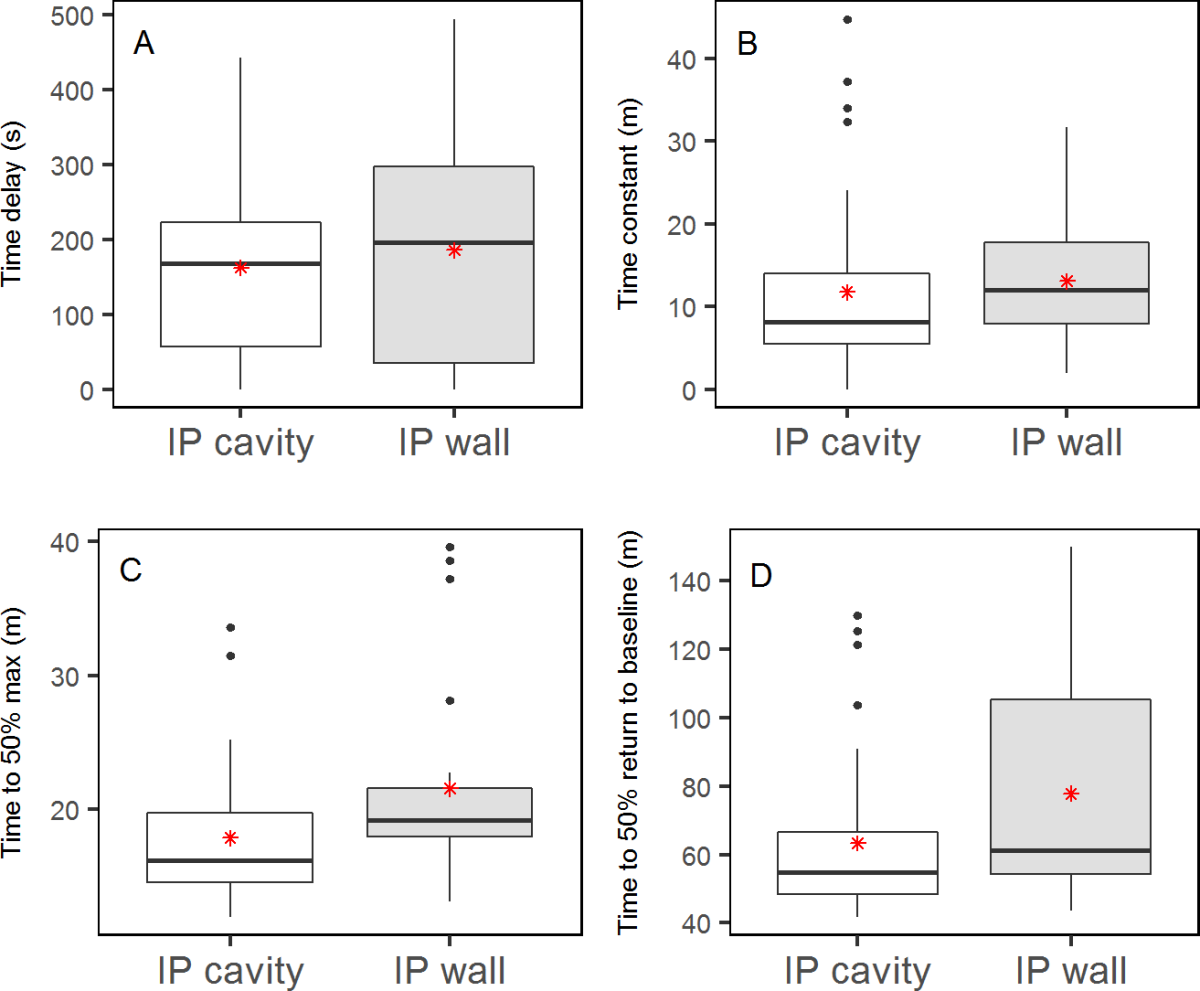
**Boxplots of time delay (A), time constant (B), time to 50% max (C) and time to 50% decline to baseline level (D) for IP sensors with different positioning of sensor element.** Mean values are presented as a red *.

The IP sensors positioned with the sensor element against the abdominal wall (75% included) show more stable signals than the sensors with the element towards the peritoneal space/visceral lining (50% included). Positioning of the sensor element also affects the glucose dynamics, being slower for the sensors facing the abdominal wall (Fig 9). The difference is, however, not statistically significant (S2 Table).

**Fig 10:**
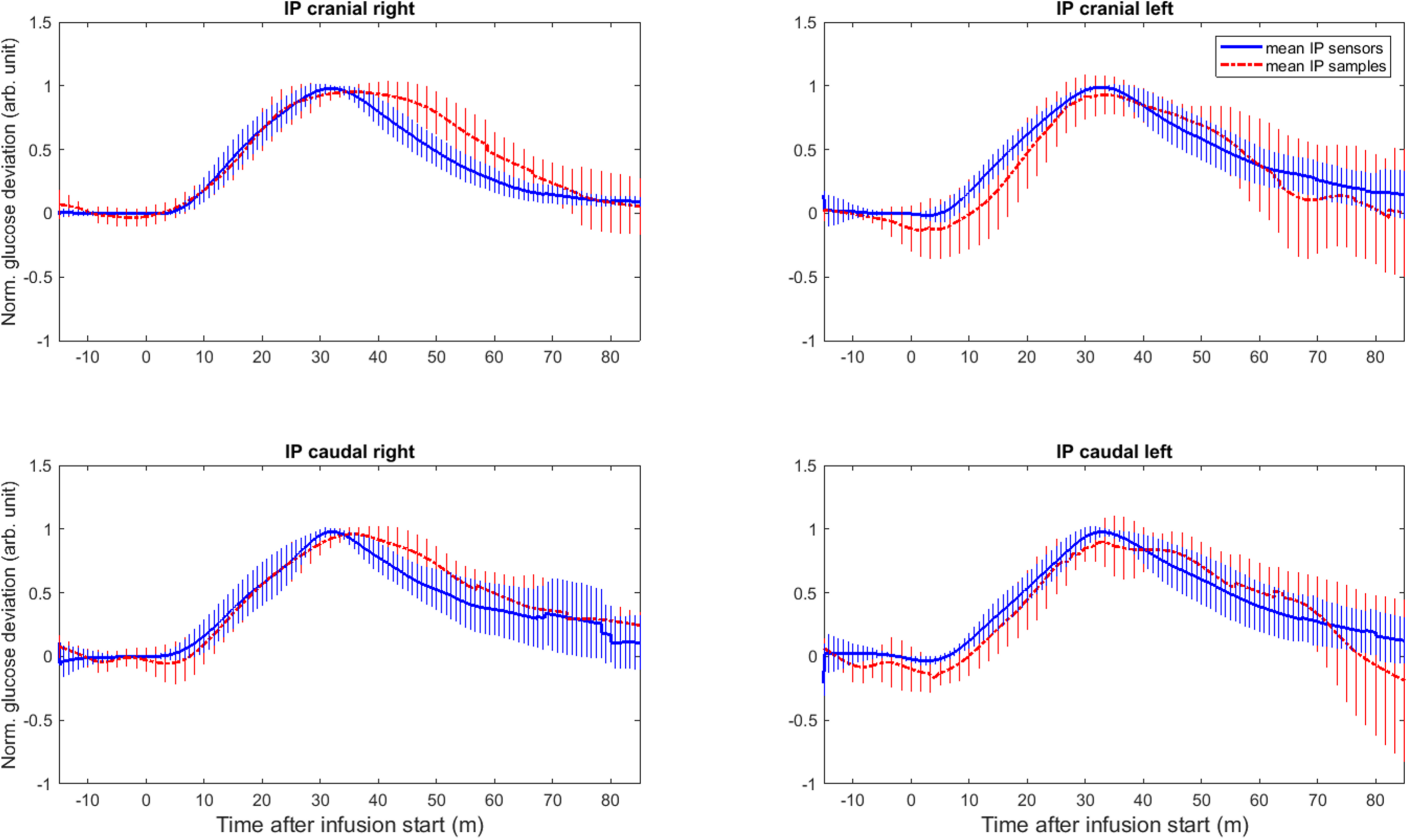
Distribution of included sensor recordings from the four different IP sensor locations compared to corresponding IP fluid samples during IV-meals. The blue curves represent the mean of the normalized sensor signals, and the red curves represent the mean of the normalized IP fluid samples. The hatched areas indicate ± 1 SD of the included data. Data was normalized by means of offset correction and scaling to the range [0, 1]. arb.unit = arbitrary unit.

IP fluid was frequently sampled during the experiments and shows increasing glucose values after IV infusions of glucose. A basic comparison with the IP sensor recordings at the same location showed no significant dynamic differences (Fig 10). The analysis using the MATLAB function *delayest* revealed time delays of 0 to 37 seconds between each IP sensor and its corresponding IP fluid samples (mean 10 s, SD 14 s). For 19 of the 30 included comparisons, the time delay was less than or equal to 2 seconds. This indicates that the internal sensor dynamics are very small compared to the physiological dynamics from blood to the peritoneal fluid. For the remaining 11 comparisons, the time delay was larger than 10 s; and the IP samples showed almost no increase. Thus, only the sensor recordings were further analysed by means of model identification.

**Fig 11:**
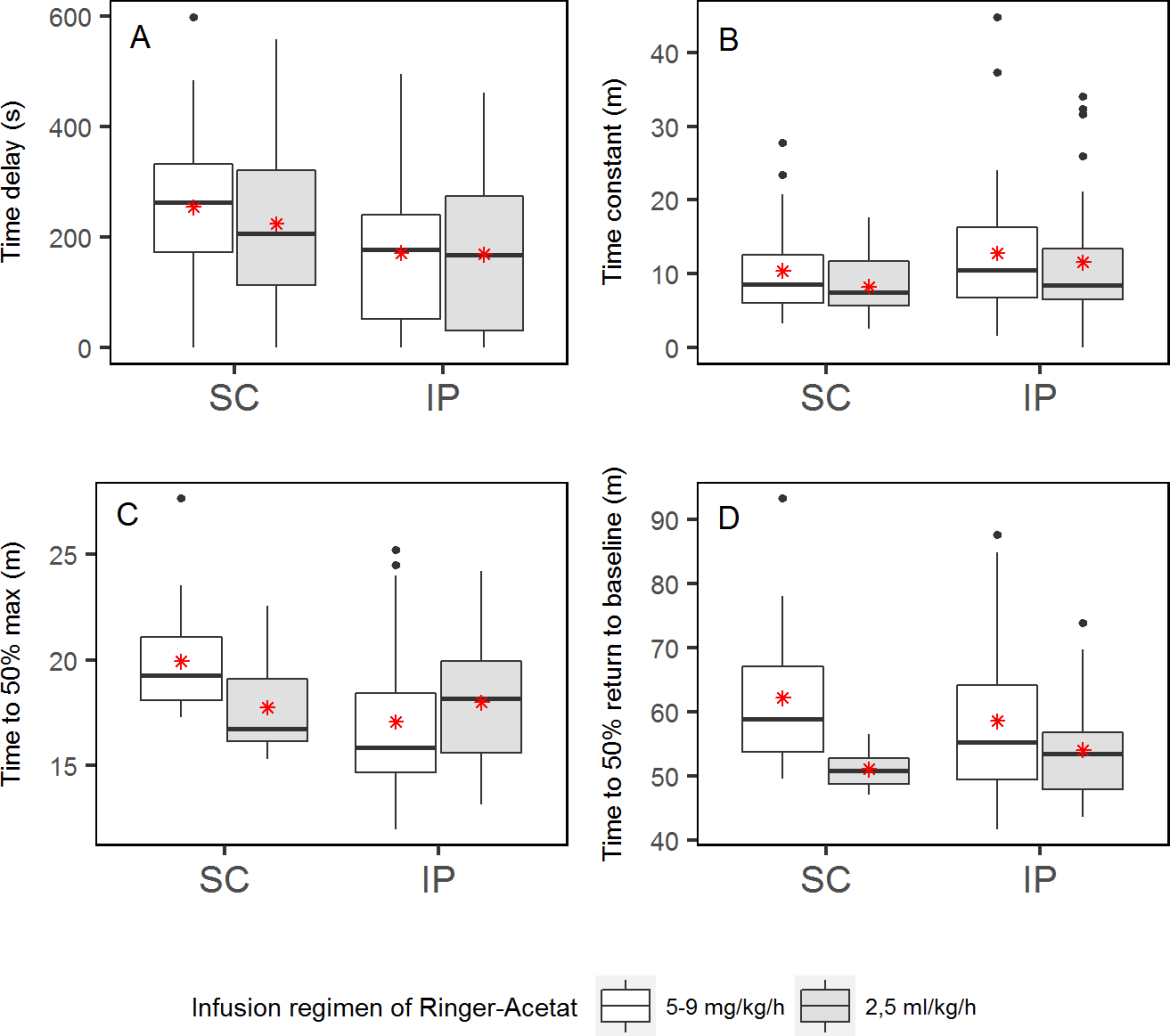
**Boxplots of time delay (A), time constant (B), time to 50% max (C) and time to 50% decline to baseline level (D) for IP sensors with the different IV fluid regimens for both IP and SC sensors.** Mean values are presented as red *.

The amount of IP fluid increased in all pigs during the experiments, i.e. during the eight hours on the operation table. Pigs 2–9 received 5–9 ml/kg/h, and pigs 10–13 received approximately 2.5 ml/kg/h of Ringer’s acetate IV. The amount of accumulated IP fluid at the end of experiments was reduced from approximately 200 ml to approximately 50 ml after changing the IV-infusion regimen. The volume of infused IV fluid affected the glucose dynamics, with the higher infusion regimen resulting in slower dynamics for both SC and IP sensors, with the exception of the time to 50% max for IP sensors (Fig 11). The differences in dynamic parameters for the two infusion regimens were not significantly different (S2 Table).

## Discussion

No obvious difference in glucose dynamics was found between four different locations in the peritoneal cavity of anaesthetised pigs. The difference in time constant between cranial left and caudal left was statistically significant. The clinical significance of this, however, is doubtful when only one of several comparisons shows statistical significance. Comparing IP sensors to SC sensors showed a significantly smaller time delay for the IP sensors, but we did not find significant results when comparing the other dynamic parameters. As secondary findings, we observed that the glucose dynamics were affected both by the amount of IV fluid given during experiments and the positioning of the sensor element (for the IP sensors), but these differences were not significant.

Glucose sensing in the peritoneal space has been studied in rabbits [30], rats [31, 32] and pigs [22, 33]. Burnett et al. reported the mean time delay and mean time constant to be 40.8 (SD 34.8) seconds and 5.6 (SD 2.9) minutes, respectively [22]. Fougner et al. have previously found a mean time delay of 9.7 (SD 9.5) seconds and mean time constant of 4.7 (SD 2.9) minutes in pigs [33]. The IP glucose dynamics (time delays and time constants) in the present investigation are slower compared to these previous studies.

Burnett et al. compared glucose sensing in the IP space to the SC tissue and found the glucose dynamics in the IP space to be significantly faster than in the SC tissue. The present investigation showed the time delay of the IP sensors to be significantly shorter than that of SC sensors. The other comparisons revealed, however, no statistically significant differences.

Comparing our present results to the previous published results is difficult for several reasons. First of all, different glucose sensors were used in the three studies: Burnett et al. used modified Dexcom sensors in their trials, which is an amperometric sensor like the FreeStyle Libre sensor, but from a different manufacturer (Dexcom, San Diego, USA). Fougner et al. used optical interferometric sensors to measure glucose [33]. Secondly, the studies handle the measured glucose dynamics in different ways. Measured glucose dynamics is composed of two parts: the glucose diffusion from the circulation to the sensing location, and the internal sensor dynamics. Fougner et al. previously used sensors with known dynamics, and identified the actual dynamics related to the sensing location [33]. In both the present study and the study by Burnett et al., the overall dynamics, i.e. the physiologic and sensor delays, are analysed together. Thus, possible differences between sensing locations may be harder to identify if the sensor has slow dynamics that hides the minor contribution of the sensing location. That way, the potential of the sensing location is not assessed, but rather the combination of the sensing location and the particular sensor. As already pointed out, internal sensor dynamics appear to be very small compared to the physiological dynamics from blood to the peritoneal fluid. Given that we could not detect differences in dynamic contributions from the different sensing locations themselves, any such contributions are also likely to be small (i.e. the resulting dynamics are fast) compared to the gross physiological dynamics from blood to peritoneal fluid. Finally, we delivered glucose as simulated meals and clamps in the presented study, while glucose was given as boluses in the previous studies. Different speeds of glucose delivery might affect the observed dynamics. It is noteworthy that in the present study, simulating physiologic glucose excursions, the overall IP glucose dynamics are slower than previously reported and not significantly faster than the SC glucose dynamics.

The peritoneal lining is made up of a single layer of mesothelial cells with an underlying layer of connective tissue embedded with capillaries, other blood vessels, nerves and lymphatic vessels [34]. According to the “three pore model” referred to in the field of peritoneal dialysis, the endothelium of the capillaries is considered to be the major barrier for water and solutes crossing the peritoneal lining [35], although the interstitium is also believed to contribute [36]. Glucose is a small molecule, and passes easily through the small pores in the endothelium, mainly by diffusion [37, 38]. It should be mentioned that the greater omentum in pigs does not cover the whole front of the intestines in the way it does in humans. The sensor elements pointing into the peritoneal space of our pigs might be positioned against the visceral peritoneal lining of the intestines, whereas they would have been facing the greater omentum in humans. However, in humans there is no systematic variation in the key histological parameters, including microvascular density, in different parts of the peritoneum [39, 40]. This fits with our observation of no evident spatial difference in glucose dynamics between the four quadrants of the peritoneal cavity. However, the observed variation in data obtained within each of the four IP quadrants might indicate local differences in glucose dynamics. More knowledge of these possibly small-scale local differences is needed, as this might lead to considerable improvements of the glucose dynamics by more accurate placing of the sensors.

The positioning of the sensor element affected both the glucose dynamics and the quality of sensor signals. The glucose dynamics of the sensors with the sensor element towards the abdominal wall was slower than the dynamics of the sensors pointing towards the visceral peritoneum (Fig 9). This might be because the sensor elements were pressed against the wall and IP fluid surrounding the sensor element was kept stagnant. The quality of sensor signals was also affected by positioning of the sensor element. The signals were less noisy when the sensor element lay against the parietal peritoneum, which could be because the sensor element was pressed against the abdominal wall in a stable position. The sensors with the element pointing inwards in the peritoneal space might be exposed to a more unstable environment of moving fluid and organs. We frequently sampled IP fluid and this most likely contributed to circulation of IP fluid. It is, however, not possible to conclude whether the fluctuating signals, especially seen in the excluded sensor recordings, are caused by actual changes in glucose concentration in IP fluid or should be considered as artefacts (e.g. due to movements of organs and IP fluid), or a combination of the two.

The Kalman smoothing we performed on the sensor data assumes dynamics by using a process model. It could be argued that the Kalman smoothing modifies the dynamics of the FreeStyle Libre data. However, the ratio of measurement to process noise was chosen low and by that, we trust the sensor values more than the process model. Our simulations showed that the smoothed glucose values follow the sensed values with good accuracy (Fig 3). The smoothing ensures that subsequent outliers that have not been eliminated by the median-filter are removed and subsequently replaced with missing values. Moreover, the original sampling intervals of the LimiTTer (reader for FreeStyle Libre sensors), vary in the range 20–22 seconds. For the identification procedure, we needed equal sampling intervals, which is one of the available output features of the Kalman smoother algorithm.

Blood glucose measurements were sampled less frequently, and they were less prone to outliers. They are interpolated assuming cubic curves. In that way, the actual blood glucose measurements are preserved and the values between the samples are estimated. We omitted the use of a Kalman smoother to better resemble the true blood glucose values: dependent on the tuning, a smoother either flattens the steep slope in the beginning of glucose infusions, or results in an overall poor fit. The interpolation, however, preserves the overall shape of the blood glucose curve. The timing of the blood glucose sampling was logged manually. This is one of the reasons why the identified time constants and time delays are not accurate to the single second.

The data was fitted to a model that has been developed to describe the SC glucose dynamics, but it has also been used previously to model IP glucose dynamics [22]. The model describes the glucose diffusion dynamics from the circulation to the sensing location. The model was fitted to the whole data curve, although the dynamics might differ between increasing and decreasing glucose concentrations. The physiologic glucose excursions in this study were slower compared to previous studies with IV glucose boluses [22, 33]. When glucose was infused as simulated meals, the pigs might have started to utilise glucose concomitantly as the IP glucose values were increasing. Thus, the two effects of glucose increasing and glucose decreasing, might overlap. The result on the model identification might be that in order to guarantee an overall good fit, the time delays and constants are overestimated to compensate for the glucose that has already been removed from the IP and SC sensing sites at the end of the glucose infusion. This effect might be less pronounced when glucose was given as boluses. A possible reason why the model achieved a higher fit for the SC sensors is that the model describes the SC dynamics better, whereas it is less suited to model the IP dynamics. However, it is out of the scope of this study to evaluate the model. Overall, Eq 1 resulted in adequately described IP glucose sensing, and it fits the purpose of this study to use the same model structure for comparison of IP and SC glucose sensing dynamics.

During several glucose infusions, the blood glucose values showed a steadily rising curve, but some IP sensors seemed to oscillate around this presumably actual value. The resulting model fit is low, although the model might actually fit the dynamics quite well. The percentage of fit of the identified models was used as an inclusion criterion. For this reason, some curves that described the actual dynamics of the sensor location well may have been excluded due to oscillations of unknown origin.

An AP requires adequate sensor technology, algorithms and an optimal site for the sensor and hormone administration. The IP space has previously been shown as a promising site for CGM due to its appearing fast glucose dynamics, but the IP glucose dynamics in the current experimental set up was equivalent to the glucose dynamics of the SC tissue. The overall advantage of IP CGM must be considered against the obvious disadvantages; entering the IP space through a port imposes a risk for a more serious infection than in the SC space. Patients may also consider the port as too invasive. Real fast glucose dynamics is therefore essential for an IP glucose sensor to be a realistic alternative for diabetic patients, and the results shown in the presented study does not justify moving the glucose sensing into the abdomen, i.e. the clinical significance of a shorter time delay in IP glucose sensing has still to be proven, and it has to be weighed against the cost of surgical efforts and patient inconvenience. However, if combined with intraperitoneal insulin delivery with an external pump, a glucose sensor can be added using the same port as the insulin tube with no additional inconvenience for the patient.

Development of alternative sensor technology might improve the performance of CGM in the peritoneal space considerably. Amperometric sensors like the FreeStyle Libre used in the presented study and the modified Dexcom sensors used by Burnett et al. [22] measure glucose in the peritoneal fluid directly surrounding and in contact with the sensor element. Glucose in the peritoneal fluid must travel from the peritoneal lining and to the location of the sensing element to be detected, and the performance of sensors measuring glucose in the peritoneal fluid will therefore be highly influenced by the amount of fluid surrounding the sensor element and the movement of the IP fluid. The diffusion coefficient of glucose in water at 25°C is 6.7 x 10^−6^ cm^2^ per second [41], and by using Fick´s second law of diffusion [42] this implies that glucose will travel 1 mm in water in approximately 750 seconds. Optical sensor technology, such as near-infrared, mid-infrared and Raman spectroscopy however, might enable glucose measurements directly on the peritoneal lining or even in the capillaries embedded in the tissue, in addition to the ability to measure in the peritoneal fluid. Fougner et al. used optical interferometric sensors in their experiments and identified fast glucose dynamics in the peritoneal space [33]. They questioned if their sensors actually measured glucose directly in contact with the capillaries in the peritoneal lining, i.e. with only a short distance, consisting of the peritoneal lining and capillary wall and sensor membrane, and suggested this as an explanation of the fast IP glucose dynamics identified in the study [33].

Nevertheless, the IP space has other advantages compared to the SC tissue; it is more protected against changes in temperature and mechanical pressure and it can prove as an acceptable solution in combination with intraperitoneal delivery of insulin, even without superior glucose dynamics compared to SC tissue.

This study has several limitations. The FreeStyle Libre sensor is designed to operate in the SC tissue. Our use of the sensor in an alternative environment might affect the presented glucose values in ways we are not fully aware of. Variations in pressure, oxygen tension, excessive moisture, bowel movements and possibly other factors might influence the measurements and cause the heavy fluctuations and unpredicted signals seen in some of the IP-sensors. These signal fluctuations are not considered to be due to disturbances in signal transmission, as digital transmission errors cause artefacts in the received signal that are qualitatively different from the ones observed here.

Unlike most other CGM devices, the FreeStyle Libre sensor is factory calibrated [43]. The sensor system presents glucose values without initial calibration by the user. Unlike some of the other CGMs, it is not possible for the user to access the raw data. The sensor is preprocessing the data before presenting the glucose value, but information about the preprocessing is not available in the open literature. The FreeStyle Libre sensor also includes a temperature sensor, likely used for a control algorithm using the temperature in the surroundings of the sensing element to adjust the glucose value. We believe this temperature adjustment might be done in the FreeStyle Libre hand held reader, and if so, the glucose values we obtain using a LimiTTer will be without this temperature correction. The glucose clamps were intended to be used for calibration of the sensors, but in this paper uncalibrated sensors recordings were used for dynamic parameter comparisons since there was a risk of the calibration method affecting the estimation of such parameters. We also observed an overshooting of the sensor response when it was exposed to large and sudden differences in glucose concentrations in in vitro trials [25]. Such sensor behaviour was not observed when the sensors were exposed to gradual increase in glucose concentration. This sensor behaviour was unexpected, and for this reason, the IV boluses were excluded from further analysis.

The experiments were performed on non-diabetic pigs, and the presented glucose dynamics can be different from a diabetic animal model, and the results are by no means directly transferable to diabetic patients. This study also faces the challenge of being conducted on anaesthetised pigs undergoing long-lasting surgical procedure. This might influence the pigs’ physiology and consequently the glucose dynamics we are examining, and is the main argument for the experiments to be of relatively short duration. For instance, we did observe an increase in IP fluid volume during the individual experiments. The total amount of IP fluid in humans or pigs is not well studied. There is no available information on the normal amount of IP fluid in pigs, and for humans the reported estimates range from 15 to 100 ml [34, 44–46]. We reduced the IV infusion rate of Ringer’s acetate (see Methods), which resulted in a reduction of produced IP fluid. However, the volumes are still suspected to be unphysiologically large. It is likely that large amounts of IP fluid during the experiments will slow both the increasing and decreasing glucose dynamics because of a diluting effect and because more fluid consequently leads to greater distances for the glucose molecules to travel before being detected by the sensor element. The sensors in the pigs receiving the lower infusion rate did react faster than the sensors in pigs receiving the higher infusion rate, but the difference was not significant. Viewing the graphical comparisons of sensor signals to sampled IP fluid (Fig 10) suggest that there is no significant difference.

Fluid also accumulated in the SC tissue in pigs receiving large volumes of IV fluid, causing slower dynamics in the SC tissue especially the time to 50% return to baseline as can be seen in Fig 11. Interestingly, this may also be the case in humans. If present, the effect is probably minor but the physiologic delays may have a diurnal variation with the lowest values in the morning before having had anything to drink. It may also increase delays in patients with heart failure. The unphysiological IP fluid volumes might make the comparisons between SC and IP sensors problematic, but we assume equal conditions for the IP sensors making the comparisons of glucose dynamics within the IP space valid.

Research on glucose sensing in the peritoneal space is at its early stage. Long-term studies will examine the glucose dynamics in awake pigs and not during prolonged anaesthesia and surgery. The amount of IP fluid will probably be normalised, the wound in the abdominal wall will have healed and the effects of anaesthesia are removed. Most importantly, however, IP glucose dynamics will be investigated during normal physiologic excursions following meals and normal fasting.

In addition to more refined and physiologic animal experiments, development of the sensor technology is crucial in examining the IP glucose dynamics. The sensor’s own dynamics should be rapid, its lifetime must be long, its sensitivity high and it should be robust.

## Conclusions

The present study found no major differences in glucose dynamics between the four quadrants of the intraperitoneal cavity of pigs. The time delay of the IP sensors was significantly smaller than that of the SC sensors (170 s vs. 241 s), but no other significant differences in subcutaneous and intraperitoneal glucose dynamics were identified.

## Supporting information

S1 Table Output from statistical analysis in R for comparisons of IP sensors. (DOCX)

S2 Table Output from statistical analysis in R for comparison of SC and IP sensors. (DOCX)
