# Supplementary material for: Effect of sensor location on continuous intraperitoneal glucose sensing in an animal model
Source: PLoS One. 2018 Oct 9;13(10):e0205447. doi: 10.1371/journal.pone.0205447 (PMC6177183; doi:10.1371/journal.pone.0205447)
Supplement: S1 Table — (DOCX) [file pone.0205447.s001.docx]

| **TimeDelay_s ~ Sensor_loc + IV_vol + TipDir + (1 \| Pig)**  **Linear mixed model fit by maximum likelihood. t-tests use Satterthwaite's method ['lmerModLmerTest']** | | | | | | | | | | | | | |
| --- | --- | --- | --- | --- | --- | --- | --- | --- | --- | --- | --- | --- | --- |
| Fixed effects |  |  |  | |  | |  | | |  | | | |
|  |  | Estimate | Std. Error | | df | | t value | | | Pr(>\|t\|) | | | |
| (Intercept) |  | 171.465 | 44.482 | | 63.00 | | 3.855 | | |  | | | |
| Cran L |  | -27.909 | 50.288 | | 63.00 | | -0.555 | | | 0.580868 | | | |
| Caud R |  | -18.238 | 52.440 | | 63.00 | | -0.348 | | | 0.729165 | | | |
| Cran L |  | 27.325 | 51.277 | | 63.00 | | 0.533 | | | 0.595980 | | | |
| IV_vol reduc |  | -5.959 | 36.963 | | 63.00 | | -0.161 | | | 0.872439 | | | |
| TipDir wall |  | 19.326 | 39.169 | | 63.00 | | 0.493 | | | 0.623447 | | | |
|  |  |  |  | |  | |  | | |  | | | |
| Groups | Name | Variance | Std.Dev. | |  | |  | | |  | | | |
| Pig | (Intercept) | 0 | 0.0 | |  | |  | | |  | | | |
| Residual |  | 18906 | 137.5 | |  | |  | | |  | | | |
| Number of obs: 63, groups: Pig, 12 | | | | | | | | | | | | | |
|  | | | | | | | | | | | | | |
| **TimeConstant_min ~ Sensor_loc + IV_vol + TipDir + (1 \| Pig)**  **Linear mixed model fit by maximum likelihood. t-tests use Satterthwaite's method ['lmerModLmerTest']** | | | | | | | | | | | | | |
| Fixed effects |  |  | |  | |  | | |  | | | |  |
|  |  | Estimate | | Std. Error | | df | | | t value | | | | Pr(>\|t\|) |
| (Intercept) |  | 13.568 | | 2.819 | | 63 | | | 4.813 | | | |  |
| Cran L |  | -4.634 | | 3.187 | | 63 | | | -1.454 | | | | 0.151 |
| Caud R |  | -1.372 | | 3.323 | | 63 | | | -0.413 | | | | 0.681 |
| Caud L |  | 2.114 | | 3.250 | | 63 | | | 0.651 | | | | 0.518 |
| IV_vol reduc |  | -1.390 | | 2.343 | | 63 | | | -0.594 | | | | 0.555 |
| TipDir wall |  | 1.172 | | 2.482 | | 63 | | | 0.472 | | | | 0.639 |
|  |  |  | |  | |  | | |  | | | |  |
| Groups | Name | Variance | | Std.Dev. | |  | | |  | | | |  |
| Pig | (Intercept) | 0 | | 0 | |  | | |  | | | |  |
| Residual |  | 75.94 | | 8.714 | |  | | |  | | | |  |
| Number of obs: 63, groups: Pig, 12 | | | | | | | | | | | | | |
|  | | | | | | | | | | | | | |
| **LibreTimeToHalfMax_min ~ Sensor_loc + IV_vol + TipDir + (1 \| Pig)**  **Linear mixed model fit by maximum likelihood. t-tests use Satterthwaite's method ['lmerModLmerTest']** | | | | | | | | | | | | | |
| Fixed effects |  |  |  | |  | |  | | | |  | | |
|  |  | Estimate | Std. Error | | Df | | t value | | | | Pr(>\|t\|) | | |
| (Intercept) |  | 17.3825 | 1.2035 | | 49 | | 14.444 | | | |  | | |
| Cran L |  | -0.9218 | 1.4129 | | 49 | | -0.652 | | | | 0.517 | | |
| Caud R |  | -0.6358 | 1.3969 | | 49 | | -0.455 | | | | 0.651 | | |
| Caud L |  | 0.1315 | 1.4028 | | 49 | | 0.094 | | | | 0.926 | | |
| IV_vol reduc |  | 0.6742 | 1.0165 | | 49 | | 0.663 | | | | 0.510 | | |
| TipDir wall |  | 0.5768 | 1.0887 | | 49 | | 0.530 | | | | 0.599 | | |
|  |  |  |  | |  | |  | | | |  | | |
| Groups | Name | Variance | Std.Dev. | |  | |  | | | |  | | |
| Pig | (Intercept) | 0.00 | 0.00 | |  | |  | | | |  | | |
| Residual |  | 11.01 | 3.317 | |  | |  | | | |  | | |
| Number of obs: 48, groups: Pig, 8 | | | | | | | | | | | | | |
|  | | | | | | | | | | | | | |
| **LibreTimeHalfDownToBaseline_min ~ Sensor_loc + IV_vol + TipDir + (1 \| Pig)**  **Linear mixed model fit by maximum likelihood. t-tests use Satterthwaite's method ['lmerModLmerTest']** | | | | | | | | | | | | | |
| Fixed effects |  |  |  | |  | | |  | | | |  | |
|  |  | Estimate | Std. Error | | df | | | t value | | | | Pr(>\|t\|) | |
| (Intercept) |  | 60.2400 | 4.2226 | | 22.8594 | | | 14.266 | | | |  | |
| Cran L |  | -4.0487 | 3.9745 | | 41.8124 | | | -1.019 | | | | 0.314 | |
| Caud R |  | -6.1675 | 3.9463 | | 40.4006 | | | -1.563 | | | | 0.126 | |
| Caud L |  | -0.1231 | 3.9688 | | 40.9038 | | | -0.031 | | | | 0.975 | |
| IV_vol reduc |  | -5.6969 | 4.6093 | | 9.9458 | | | -1.236 | | | | 0.245 | |
| TipDir wall |  | 3.3870 | 3.6655 | | 46.0092 | | | 0.924 | | | | 0.360 | |
|  |  |  |  | |  | | |  | | | |  | |
| Groups | Name | Variance | Std.Dev. | |  | | |  | | | |  | |
| Pig | (Intercept) | 24.58 | 4.958 | |  | | |  | | | |  | |
| Residual |  | 83.12 | 9.117 | |  | | |  | | | |  | |
| Number of obs: 47, groups: Pig, 8 | | | | | | | | | | | | | |
