# Supplementary material for: Effect of sensor location on continuous intraperitoneal glucose sensing in an animal model
Source: PLoS One. 2018 Oct 9;13(10):e0205447. doi: 10.1371/journal.pone.0205447 (PMC6177183; doi:10.1371/journal.pone.0205447)
Supplement: S2 Table — (DOCX) [file pone.0205447.s002.docx]

| **TimeDelay_s ~ SC_IP + IV_vol + (1 \| Pig)**  **Linear mixed model fit by maximum likelihood. t-tests use Satterthwaite's method ['lmerModLmerTest']** | | | | | | | | | | |
| --- | --- | --- | --- | --- | --- | --- | --- | --- | --- | --- |
| Fixed effects |  |  |  |  |  | |  | | | |
|  |  | Estimate | Std. Error | df | t value | | Pr(>\|t\|) | | | |
| (Intercept) |  | 247.64 | 22.31 | 119 | 11.099 | |  | | | |
| IP |  | -70.13 | 26.45 | 119 | -2.651 | | 0.00912 | | | |
| IV_vol reduc |  | -15.21 | 26.48 | 119 | -0.574 | | 0.56692 | | | |
|  |  |  |  |  |  | |  | | | |
| Groups | Name | Variance | Std.Dev. |  |  | |  | | | |
| Pig | (Intercept) | 2.112e-10 | 1.453e-05 |  |  | |  | | | |
| Residual |  | 2.066e+04 | 1.437e+02 |  |  | |  | | | |
| Number of obs: 119, groups: Pig, 12 | | | | | | | | | | |
| **TimeConstant_min ~ SC_IP + IV_vol + (1 \| Pig)**  **Linear mixed model fit by maximum likelihood. t-tests use Satterthwaite's method ['lmerModLmerTest']** | | | | | | | | | | |
|  | | | | | | | | | | |
| Fixed effects |  |  |  |  | |  | | | |  |
|  |  | Estimate | Std. Error | df | | t value | | | | Pr(>\|t\|) |
| (Intercept) |  | 10.212 | 1.202 | 31.392 | | 8.497 | | | |  |
| IP |  | 2.796 | 1.375 | 111.604 | | 2.013 | | | | 0.0465 |
| IV_vol reduc |  | -1.664 | 1.472 | 10.162 | | -1.131 | | | | 0.2840 |
|  |  |  |  |  | |  | | | |  |
| Groups | Name | Variance | Std.Dev. |  | |  | | | |  |
| Pig | (Intercept) | 0.6617 | 0.8134 |  | |  | | | |  |
| Residual |  | 55.7761 | 7.4683 |  | |  | | | |  |
| Number of obs: 119, groups: Pig, 12 | | | | | | | | | | |
|  | | | | | | | | | | |
| **LibreTimeToHalfMax_min ~ SC_IP + IV_vol + (1 \| Pig)**  **Linear mixed model fit by maximum likelihood. t-tests use Satterthwaite's method ['lmerModLmerTest']** | | | | | | | | | | |
| Fixed effects |  |  |  |  |  | | |  | | |
|  |  | Estimate | Std. Error | Df | t value | | | Pr(>\|t\|) | | |
| (Intercept) |  | 19.1368 | 0.6766 | 13.7570 | 28.282 | | |  | | |
| IP |  | -1.4330 | 0.6606 | 77.3438 | -2.169 | | | 0.0331 | | |
| IV_vol reduc |  | -0.4348 | 0.8189 | 7.5300 | -0.531 | | | 0.6107 | | |
|  |  |  |  |  |  | | |  | | |
| Groups | Name | Variance | Std.Dev. |  |  | | |  | | |
| Pig | (Intercept) | 0.4817 | 0.6941 |  |  | | |  | | |
| Residual |  | 9.0148 | 3.0025 |  |  | | |  | | |
| Number of obs: 85, groups: Pig, 8 | | | | | | | | | | |
|  | | | | | | | | | | |
| **LibreTimeHalfDownToBaseline_min ~ SC_IP + IV_vol + (1 \| Pig)**  **Linear mixed model fit by maximum likelihood. t-tests use Satterthwaite's method ['lmerModLmerTest']** | | | | | | | | | | |
| Fixed effects |  |  |  |  |  | | | |  | |
|  |  | Estimate | Std. Error | Df | t value | | | | Pr(>\|t\|) | |
| (Intercept) |  | 59.6534 | 2.8586 | 10.8315 | 20.868 | | | |  | |
| IP |  | -0.5569 | 1.9457 | 75.6771 | -0.286 | | | | 0.775 | |
| IV_vol reduc |  | -6.7282 | 3.7817 | 8.3378 | -1.779 | | | | 0.112 | |
|  |  |  |  |  |  | | | |  | |
| Groups | Name | Variance | Std.Dev. |  |  | | | |  | |
| Pig | (Intercept) | 21.03 | 4.586 |  |  | | | |  | |
| Residual |  | 76.51 | 8.747 |  |  | | | |  | |
| Number of obs: 83, groups: Pig, 8 | | | | | | | | | | |
